# Is longer axial length protective of vision-threatening diabetic retinopathy across different ages? A multicenter cohort of 736 patients

**DOI:** 10.1186/s40942-024-00593-x

**Published:** 2024-10-10

**Authors:** Mingpeng Xu, Bo Li, Chenxin Li, Peiwei Chai, Qinghua Qiu, Zhi Zheng, Qian Chen, Dawei Luo, Xiaofang Xu, Chuandi Zhou

**Affiliations:** 1 Department of Ophthalmology, Xinhua Hospital, Shanghai Jiao Tong University School of Medicine, Shanghai, China; 2 Department of Ophthalmology, The Fourth Affiliated Hospital of Soochow University, Suzhou, Jiangsu China; 3 Department of Ophthalmology, Shanghai General Hospital, National Clinical Research Center for Eye Diseases, Shanghai Jiao Tong University School of Medicine, No. 100 Haining Road, Hongkou District, Shanghai, 200080 China; 4 Department of Ophthalmology, Ninth People’s Hospital, Shanghai Jiao Tong University School of Medicine, No. 639 Zhizaoju Road, Shanghai, 200011 China; 5 Department of Ophthalmology, Tongren Hospital, Shanghai Jiao Tong University School of Medicine, No. 1111 Xianxia Road, Changning District, Shanghai, 200336 China

**Keywords:** Diabetic retinopathy, Axial length, Tractional retinal detachment, Best-corrected visual acuity, Neovascular glaucoma, Recurrent vitreous hemorrhage

## Abstract

**Purpose:**

Vision-threatening diabetic retinopathy (VTDR) included severe non-proliferative diabetic retinopathy (NPDR), proliferative diabetic retinopathy (PDR) and clinically significant diabetic macular edema (DME). To compare the axial length (AL) and assess its influence on VTDR across different ages.

**Methods:**

A retrospective cohort study. Medical chart review was performed in 736 consecutive patients with VTDR. The patients were divided into young (≤ 45 years) and elderly group (> 45 years) based on their age at the diagnosis of VTDR. After at least one year of standardized treatments, all eligible patients were followed up. The main outcome measures included the presence of tractional retinal detachment (TRD) involving foveal, final best-corrected visual acuity (BCVA), the development of neovascular glaucoma (NVG), and recurrent vitreous hemorrhage (VH) post-vitrectomy. ALs were compared between two age groups. The impact of AL on clinical outcomes was determined by logistic analyses after controlling for systemic parameters.

**Results:**

The study included 144 patients ≤ 45 years and 592 patients > 45 years. Young patients had significantly longer AL than elderly participants (23.9 mm vs 23.0 mm, p < 0.001). Over a median follow-up of 25.9 months, a larger proportion of young patients developed TRD (34.7% vs 16.2%, p < 0.001) and recurrent VH (18.6% vs 10.3%, p = 0.040) than elderly patients. In elderly group, longer AL is an independent protective factor in preventing TRD (odds ratio [OR], 0.5; 95% confidence interval [CI], 0.4–0.7; P < 0.001). However, this beneficial effect was not observed in young patients.

**Conclusions:**

Young patients with VTDR exhibited significantly longer AL but more aggressive clinical signs with compromised prognosis. In elderly group, a longer AL independently reduced the risk of TRD, while this protective effect did not exist for young patients.

## Introduction

Diabetic retinopathy (DR) is the leading cause of blindness in working age adults [1]. Approximately one third of DR patients develop vision-threatening changes [2], including severe non-proliferative DR (NPDR), proliferative DR (PDR) and clinically significant diabetic macular edema (DME) [1]. The progression of DR features worsens with increasing severity; it begins with mild non-proliferative abnormalities in the retina, which are often insidious and do not affect central vision initially. These early changes, typically marked by microaneurysms, can progress to exudative changes and macular edema. As ischemic conditions intensify, they may lead to PDR, and generally remain undetected until they escalate into vision-threatening diabetic retinopathy (VTDR) [3, 4]. Early detection and intervention are crucial and can prevent up to 98% of visual loss associated with DR [5], emphasizing the need for regular monitoring to catch these changes before they become severe.

Substantial evidence supports the protective effect of long AL on DR [6–9]. In addition, longer AL predicts better anatomical and functional outcome after vitrectomy [8, 9]. However, controversial remains on the effect of AL for VTDR [6, 7, 10, 11]. Few studies have investigated the association of AL with the manifestations and surgical results of VTDR, such as, the incidence of tractional retinal detachment (TRD) and neovascular glaucoma (NVG), as well as, recurrent vitreous hemorrhage (VH) post-vitrectomy.

Interestingly, a previous report revealed that young patients with DR tended to have longer AL than patients 60 years or older [7]. Meanwhile, the clinical presentations and visual outcomes among patients with VTDR differ greatly by age [12, 13]. Compared with elderly patients, proliferative impairments and rapidly declined vision were more frequently reported in young individuals [13]. Consequently, the impact of AL on VTDR varies across different ages.

In this large retrospective study, we followed up 736 patients of VTDR who had received standardized treatments for at least 1 year, to compare the AL and assess its influence on vision-threatening diabetic retinopathy (VTDR) with relation to disease severity, visual outcomes and postoperative complications across different ages after adjusting for systemic parameters.

## Methods

### Patients

Medical records were reviewed for consecutive patients initially diagnosed with VTDR in the Department of Ophthalmology, Ninth People’s Hospital, Xinhua Hospital and Shanghai General Hospital affiliated to Shanghai Jiao Tong University School of Medicine from January 2018 to December 2021. The severity of DR was scaled according to the Early Treatment Diabetic Retinopathy Study (ETDRS) grading standards [14]: mild-moderate NPDR (ETDRS level 20–47), severe NPDR (ETDRS level 53), and PDR (ETDRS level ≥ 60). Clinically significant DME was considered as retinal edema or hard exudates approaching or involving the fovea [15], confirmed by optical coherence tomography (OCT) [16, 17]. VTDR included severe NPDR, PDR and clinically significant DME. The exclusion criteria were as follows: (1) follow-up period < 12 months [18]; (2) inability to adhere to standardized treatments due to economic, geographic, or other reasons; (3) severe visual impairments other than DR, such as neovascular age-related macular degeneration, uveitis, primary glaucoma, and no light perception in one or both eyes; (4) both eyes were affected by TRD involving the fovea or other causes resulted in the axial length being unobtainable; and (5) incomplete data collection.

All patients underwent one or more of the following treatments: pan-retinal photocoagulation (PRP), intravitreal injections of anti-vascular endothelial growth factor (VEGF) agents, intravitreal injection of dexamethasone implant, or pars plana vitrectomy (PPV). PRP was indicated for eyes with severe NPDR and PDR. Intravitreal injection of anti-VEGF agents was administered to the eyes with clinically significant DME or those with active fibrovascular proliferation, scheduled for vitrectomy. Intravitreal dexamethasone implant was reserved for the refractory cases of DME to conventional treatments. The indications for vitrectomy were non-clearing VH lasting more than one month and/or TRD involving the foveal.

Informed consent was obtained from each patient. This study adhered to the tenets of the Declaration of Helsinki and received approval from the Institutional Review Board of Shanghai General Hospital, affiliated with Shanghai Jiao Tong University School of Medicine (identifier, 2022KY024, Supporting file 1). All the patients were fully informed and participated in this study voluntarily without additional compensation.

### Data collection

The baseline was set at the date of the diagnosis of VTDR. Data collected included demographics, clinical characteristics and clinical outcomes at follow-up. The demographics consisted of gender, age and educational level. Patients were stratified into two groups as young (≤ 45 years old) and elderly group (> 45 years old) based on their age at the diagnosis of VTDR [19]. Baseline ocular data were recorded, including best-corrected visual acuity (BCVA) tested with a Snellen chart, spherical equivalent, AL examined by IOLMaster 700 (Carl Zeiss Meditec AG, Jena, Germany). For eyes with foveal-involved tractional detachment, the AL of the contralateral eye was used if the patient without anisometropia. In addition, posterior vitreous detachment (PVD) status was assessed intraoperatively for patients underwent PPV. Complete PVD was considered as separation of the posterior hyaloid from both the macula and optic nerve. Systemic parameters obtained from the electronic chart records were duration of diabetes, the presence of diabetic nephropathy (DN) (albumin/urine creatinine ratio ≥ 30 mg/g), smoking status, systolic and diastolic blood pressure, body mass index (BMI), and biochemistry laboratory information on glycated hemoglobin (HbA1c) and low-density lipoprotein (LDL) cholesterol. Included variables were assessed every 3–6 months. The last information before the diagnosis of VTDR was carried forward. For patients with bilateral VTDR, the eye with worse BCVA was selected for analyses, whereas for both eyes with the same BCVA, we selected one eye randomly for analysis. Major clinical outcomes documented were presence of TRD involving foveal, NVG, final BCVA at follow-up < 0.3 (decimal visual acuity), and recurrent VH post-vitrectomy.

### Statistical analysis

BCVA was converted to the logarithm of the minimum angle of resolution (logMAR). Counting fingers, hand motion and light perception were assigned the logMAR units of 2.1, 2.4 and 2.7,

respectively. The data were analyzed using IBM SPSS Statistics 25.0 (SPSS, Inc., Chicago, IL). Frequency (percentage), mean (standard deviations) and median (interquartile range) were reported for the description of categorical variables and continuous variables with normal and skewed distribution, respectively. Means, medians and proportions were compared using the student’s *t*-test, nonparametric Mann–Whitney *U* test and the chi-square test (or Fisher exact test, if appropriate), respectively. Univariate and multivariate logistic regression was performed to investigate the association between AL and major outcomes. A two-sided p-value < 0.05 was considered statistically significant.

## Results

The study included 736 patients (736 eyes), among them, 144 patients were ≤ 45 years (median age: 37.5 years) and 592 were > 45 years (median age: 59.0 years). Baseline clinical characteristics of both groups are summarized in Table 1. Compared with elderly patients, the young patients have longer AL (23.9 mm vs 23.0 mm, p < 0.001), higher myopia (-2.1D vs -0.6D, p < 0.001), a shorter duration of DM (10.0 years vs 16.0 years, p < 0.001), a higher male ratio (65.3% vs 55.7%, p = 0.038), a higher educational level (college school or higher: 29.2% vs 3.7%, p < 0.001), a lower type 2 diabetes ratio (77.8% vs 98.0%, p < 0.001), lower systolic blood pressure (125.5 mmHg vs 133.5 mmHg, p < 0.001), higher diastolic blood pressure (81.7 mmHg vs 79.1 mmHg, p < 0.001) and higher BMI (24.8 kg/m^2^ vs 23.9 kg/m^2^, p < 0.001). No significant difference was found in HbA1c (p = 0.092), LDL (p = 0.867), smoking (p = 0.246), and the presence of DN (p = 0.788). Additionally, within a cohort of 86 young patients who underwent PPV, complete PVD was observed in only 4 (4.7%) cases. This incidence is lower compared to that observed in elderly patients, where among 273 patients subjected to PPV, 24 (8.8%) exhibited complete PVD. However, this difference did not achieve statistical significance (p = 0.253; not presented in the table).

**Table 1:** Demographics and baseline clinical characteristics by age at the diagnosis of VTDR

	Total (n = 736)	≤ 45 years (n = 144)	> 45 years (n = 592)	P
Age, year	55.3 (48.0,64.0)	37.5 (33.0,42.0)	59.0 (54.0,65.0)	< 0.001*
Male gender	424 (57.6)	94 (65.3)	330 (55.7)	0.038*
Duration of diabetes, year	15.1 (10,20)	10.0 (5.0,15.0)	16.0 (10.0,23.0)	< 0.001*
College school or higher	64 (8.7)	42 (29.2)	22 (3.7)	< 0.001*
Axial length, mm	23.34 (22.5,23.9)	23.9 (23.1,24.9)	23.0 (22.4,23.7)	< 0.001*
Refractive Error, diopter	− 0.9 (− 0.5,0.0)	− 2.1 (− 4.0,0.0)	− 0.6 (0.0,0.0)	< 0.001*
Type of diabetes				<0.001*
Type 1 diabetes	44 (6.0)	32 (22.2)	12 (2.0)	
Type 2 diabetes	692 (94.0)	112 (77.8)	580 (98.0)	
HbAlc, %	7.7 (7.0,8.0)	7.6 (6.7,8.0)	7.7 (7.0,8.0)	0.092
SBP, mmHg	133.5 (122,143)	125.5 (115.0,140.0)	133.5 (124.0,145.0)	< 0.001*
DBP, mmHg	79.6 (74.0,85.8)	81.7 (75.0,89.8)	79.1 (73.0,85.0)	< 0.001*
LDL, mmol/L	2.7 (2.1,3.1)	2.7 (2.0,3.2)	2.8 (2.1,3.1)	0.867
BMI, kg/m^2^	24.2 (21.9,26.0)	24.8 (22.4,27.6)	23.9 (21.7,25.7)	< 0.001*
With diabetic nephropathy	422 (57.3)	84 (58.3)	338 (57.1)	0.788
Current smoker	236 (32.1)	52 (36.1)	184 (31.1)	0.246

Data are presented as median (interquartile range) or number (%)

VTDR, vision-threatening diabetic retinopathy; SBP, systolic blood pressure; DBP, diastolic blood pressure; LDL, low density lipoprotein; BMI, body mass index

^*^Statistically significant

After a median follow-up of 25.9 months, larger proportion of young patients (50, 34.7%) developed TRD involving foveal, which is significantly higher than that of elderly patients (96, 16.2%, p < 0.001). Of 86 eyes underwent vitrectomy in young group, 16 (18.6%) had recurrent VH, a notably higher chances than that in the elderly group (10.3%, p = 0.040). However, no significant difference was observed in the proportion of patients with final BCVA < 0.3 (59.0% vs 55.4%, p = 0.432) or with the development of NVG (7.6% vs 8.3%, p = 0.802) between two age groups (shown in Table 2).

**Table 2:** Clinical outcomes after at least 1-year standardized treatments for VTDR

	Total	≤ 45 years	> 45 years	P
TRD involving foveal (n = 736)	146 (19.8%)	50 (34.7%)	96 (16.2%)	< 0.001*
Final BCVA < 0.3 (n = 736)	413 (56.1%)	85 (59.0%)	328 (55.4%)	0.432
Recurrent VH (n = 359)	44 (12.3%)	16 (18.6%)	28 (10.3%)	0.040*
NVG (n = 736)	60 (8.2%)	11 (7.6%)	49 (8.3%)	0.802

Data presented are number (%)

VTDR, vision-threatening diabetic retinopathy; TRD, tractional retinal detachment; BCVA, best-corrected visual acuity; NVG, neovascular glaucoma

^*^Statistically significant

**Fig. 1:**
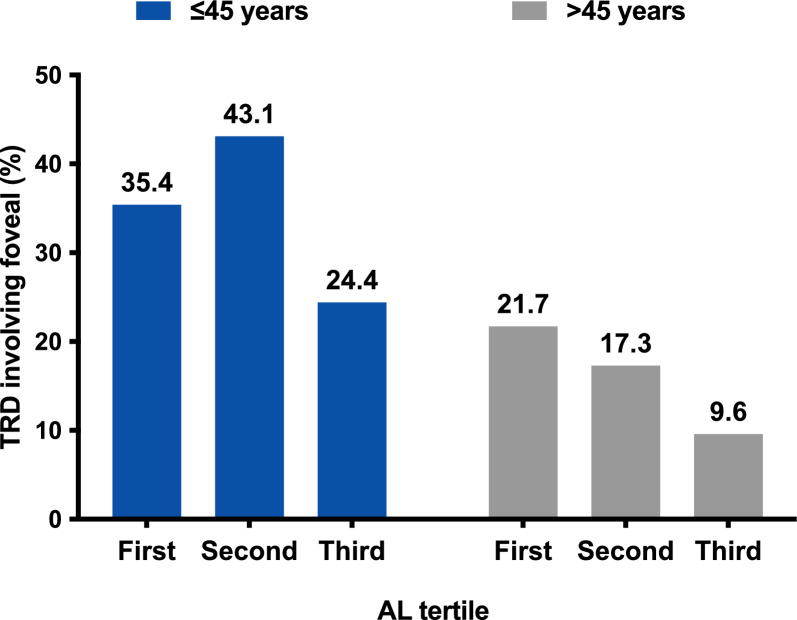
Evaluation of TRD Incidence by AL Across Age Groups in VTDR Patients. This figure contrasts the incidence of TRD among patients with VTDR, segmented into two primary age groups: ≤ 45 years and > 45 years. For the ≤ 45 years age cohort, the bars demonstrate that the incidence of TRD across varying AL tertiles—first tertile at 35.4%, second tertile at 43.1%, and third tertile at 24.4%—does not establish a definitive correlation between AL and TRD risk. This observation suggests that among younger VTDR patients, AL may not be a significant factor influencing the occurrence of TRD. In contrast, for patients older than 45 years (first tertile at 21.7%, second tertile at 17.3%, and third tertile at 9.6%), a noticeable trend indicates a reduction in TRD incidence with an increase in AL, implying that a longer AL could afford a protective advantage against TRD in the older population

The tertile distribution of AL was assessed separately for patients aged 45 years and younger, and for those older than 45 years. After categorizing the AL values in patients > 45 years old, there was a trend that the chances of TRD decreased with longer AL (first tertile: 21.7%, second tertile: 17.3%, and third tertile: 9.6%). A significant protective effect was associated with the highest AL tertile in preventing TRD compared to the lowest AL tertile as shown in Table 3 (OR = 0.4; 95%CI:0.2–0.7; p = 0.002; p for trend = 0.001). However, this association was not evident in young group (OR, 0.8; 95%CI: 0.6–1.1; p = 0.204; p for trend = 0.281) (Fig. 1). Furthermore, a longer AL did not confer any protective effect against low vision (final BCVA < 0.3) (≤ 45 years: p = 0.709; > 45 years: p = 0.291), recurrent VH following PPV (≤ 45 years: p = 0.705; > 45 years: p = 0.870) or the development of NVG (≤ 45 years: p = 0.285; > 45 years: p = 0.475) in patients regardless of age.

**Table 3:** The association of axial length and clinical characteristics of VTDR in unadjusted models

	Axial length	≤ 45 years			> 45 years		
		No. (%)	OR (95%CI)	P	No. (%)	OR (95%CI)	P
TRD involving foveal	First tertile	17 (35.4%)	Ref		43 (21.7%)	Ref	
	Second tertile	22 (43.1%)	1.4 (0.6,3.1)	0.433	34 (17.3%)	0.8 (0.5,1.2)	0.264
	Third tertile	11 (24.4%)	0.6 (0.2,1.5)	0.251	19 (9.6%)	0.4 (0.2,0.7)	0.001*
	For trend		0.8 (0.5,1.2)	0.281		0.6 (0.5,0.8)	0.001*
	Per mm increase		0.8 (0.6,1.1)	0.204		0.7 (0.6,0.9)	0.002*
Final BCVA < 0.3	First tertile	26 (54.2%)	Ref		117 (59.1%)	Ref	
	Second tertile	33 (64.7%)	1.6 (0.7,3.5)	0.287	105 (53.3%)	0.8 (0.5,1.2)	0.246
	Third tertile	26 (57.8%)	1.2 (0.5,2.6)	0.726	106 (53.8%)	0.8 (0.5,1.2)	0.290
	For trend		1.1 (0.7,1.6)	0.709		0.9 (0.7,1.1)	0.291
	Per mm increase		0.9 (0.7,1.2)	0.579		0.9 (0.8,1.0)	0.156
Recurrent VH	First tertile	7 (24.1%)	Ref		11 (10.5%)	Ref	
	Second tertile	4 (12.1%)	0.4 (0.1,1.7)	0.224	8 (9.0%)	0.8 (0.3,2.2)	0.729
	Third tertile	5 (20.8%)	0.8 (0.2,3.0)	0.775	9 (11.4%)	1.1 (0.4,2.8)	0.843
	For trend		0.9 (0.4,1.8)	0.705		1.0 (0.6,1.7)	0.870
	Per mm increase		1.0 (0.6,1.7)	0.979		1.1 (0.8,1.5)	0.716
NVG	First tertile	5 (10.4%)	Ref		19 (9.6%)	Ref	
	Second tertile	4 (7.8%)	0.7 (0.2,2.9)	0.657	15 (7.6%)	0.8 (0.4,1.6)	0.483
	Third tertile	2 (4.4%)	0.4 (0.1,2.2)	0.289	15 (7.6%)	0.8 (0.4,1.6)	0.483
	For trend		0.6 (0.3,1.4)	0.285		0.9 (0.6,1.3)	0.475
	Per mm increase		0.7 (0.4,1.3)	0.317		1.0 (0.8,1.2)	0.851

VTDR, vision-threatening diabetic retinopathy; OR, odds ratios; CI: confidence interval; TRD, tractional retinal detachment; BCVA, best-corrected visual acuity; NVG, neovascular glaucoma; VH, vitreous hemorrhage

^*^Statistically significant

The above associations persisted after additional adjustment for systemic factors including age, gender, smoking status, duration of DM, HbA1c, LDL, systolic blood pressure, BMI, and the presence of DN, type of diabetes as shown in Table 4. In elderly patients, a longer AL was an independent protective factor in preventing TRD (OR, 0.5; 95%CI, 0.4–0.7, p for trend < 0.001). The risk of having TRD decreased by 40% for each millimeter increase in AL (OR, 0.6; 95%CI: 0.5–0.8; p < 0.001). However, no remarkable association was identified for low vision (final BCVA < 0.3) (≤ 45 years: p = 0.341; > 45 years: p = 0.455), recurrent VH (≤ 45 years: p = 0.422; > 45 years: p = 0.550) or developing NVG (≤ 45 years: p = 0.579; > 45 years: p = 0268) in either age groups.

**Table 4:** The impact of axial length on VTDR adjusted for systemic parameters ^a^

	Axial length (mm)	≤ 45 years		> 45 years	
		OR (95%CI)	P	OR (95%CI)	P
TRD involving foveal	First tertile	Ref		Ref	
	Second tertile	1.7 (0.6,4.6)	0.325	0.6 (0.4,1.1)	0.12
	Third tertile	0.5 (0.2,1.6)	0.251	0.3 (0.1,0.5)	< 0.001*
	For trend	0.7 (0.4,1.3)	0.273	0.5 (0.4,0.7)	< 0.001*
	Per mm increase	0.9 (0.6,1.2)	0.40	0.6 (0.5,0.8)	< 0.001*
Final BCVA < 0.3	First tertile	Ref		Ref	
	Second tertile	1.8 (0.7,4.7)	0.199	0.8 (0.5,1.2)	0.294
	Third tertile	1.6 (0.6,4.4)	0.328	0.8 (0.5,1.3)	0.432
	For trend	1.3 (0.8,2.1)	0.341	0.9 (0.7,1.1)	0.455
	Per mm increase	1.0 (0.7,1.4)	0.870	0.9 (0.8,1.1)	0.272
Recurrent VH	First tertile	Ref		Ref	
	Second tertile	0.2 (0.03,1.8)	0.161	0.8 (0.3,2.4)	0.725
	Third tertile	0.4 (0.05,3.9)	0.447	0.7 (0.2,2.2)	0.552
	For trend	0.6 (0.2,2.0)	0.422	0.8 (0.5,1.5)	0.550
	Per mm increase	0.7 (0.2,2.0)	0.497	1.0 (0.6,1.5)	0.915
NVG	First tertile	Ref		Ref	
	Second tertile	0.8 (0.1,4.9)	0.815	0.7 (0.3,1.6)	0.398
	Third tertile	0.5 (0.05,5.3)	0.574	0.6 (0.3,1.4)	0.265
	For trend	0.7 (0.2,2.2)	0.579	0.8 (0.5,1.2)	0.268
	Per mm increase	0.9 (0.4,1.9)	0.796	0.9 (0.7,1.2)	0.657

VTDR, vision-threatening diabetic retinopathy; OR, odds ratios; CI: confidence interval; TRD, tractional retinal detachment; BCVA, best-corrected visual acuity; NVG, neovascular glaucoma; VH, vitreous hemorrhage

^a^Adjusted systemic parameters included age, gender, smoking, type of diabetes, duration of diabetes, HbA1c, low density lipoprotein, systolic blood pressure, body mass index, and the presence of diabetic nephropathy, and the presence of complete posterior vitreous detachment

^*^Statistically significant

## Discussion

This retrospective cohort study revealed that AL was significantly longer in young patients with VTDR as compared with elderly patients. However, young patients with VTDR exhibited more aggressive clinical signs and worse prognosis. In addition, longer AL served as an independent protective factor against developing TRD in elderly patients; however, this protective effect was not prominent in young patients. Nevertheless, AL was not a dominant influencing factor for visual recovery, the development of NVG, or relapsed VH.

AL has known to be a protective factor of mild and moderate DR, however, controversial remains for its role in VTDR. In a cross-sectional, population-based study, He et al., revealed each millimeter increase in AL reduced the chances of any DR and moderate DR by 12% and 11%, respectively, and yet no beneficial effect was found for VTDR [7]. Man et al., conducted a cross-sectional clinic-based study, and demonstrated that eyes with longer AL have lesser risk of mild, moderate DR as well as VTDR [6]. Another population-based, cross-sectional study confirmed the beneficial effect of longer AL in preventing all severities of DR. More specifically, this effect was most prominent for VTDR, in which, longer AL could sharply reduce the risk by 37% [11]. The impact of AL on anatomical and visual outcomes after diabetic vitrectomy has also been explored. Wakabayashi et al., conducted a cohort study of 41 eyes with non-tractional DME, and showed that longer AL predicted better vision recovery and faster restoration of the inner and outer segment (IS/OS) line after vitrectomy [8]. Song et al., revealed that longer AL was a significant predictor for anatomical success after vitrectomy, possibly due to more complete posterior vitreous detachment in longer eyes [9]. In contrast, Kim et al., followed up 24 PDR patients [26 eyes] with tractional retinal elevation, and found that eyes with longer AL were more likely to develop TRD, possibly attributable to more movable vitreous in a larger vitreous cavity [10]. Potential explanations for the discrepancies among these studies may lie in varying study designs, different patient selections, and dissimilar sample sizes. More importantly, the prognostic factors of VTDR are multifactorial, consisting of disease-, patient-, and treatment-related parameters, AL could hardly change the clinical presentations and surgical outcome independently.

Our study revealed that younger patients with VTDR exhibited more aggressive clinical signs and a higher incidence of complications such as TRD and recurrent VH, despite having a longer AL compared to elderly patients. Interestingly, while a longer AL was a protective factor against TRD in elderly patients, this effect was not observed in younger individuals. This discrepancy may be attributed to the higher prevalence of type 1 diabetes among younger patients in our cohort, as type 1 diabetes is associated with a more rapid progression of DR and a greater propensity for severe complications, including TRD and VH [20]. Studies have shown that younger patients with type 1 diabetes, particularly those with poor glycemic control and longer diabetes duration, face a significantly higher risk of developing PDR and related complications, such as NVG [21]. Additionally, the progression of DR in younger patients is often exacerbated by genetic predispositions, socioeconomic challenges, and other modifiable risk factors, further complicating their prognosis [22]. In contrast, in older patients with type 2 diabetes, the protective effect of longer AL against TRD has been observed, aligning with previous findings that suggest AL may play a more significant role in reducing the severity of DR in this demographic [23]. However, the lack of a significant protective effect of AL in younger patients may be related to the more aggressive course of DR in type 1 diabetes, as reported in various studies [24]. These findings underscore the importance of considering both age and diabetes type when assessing the risk and prognosis of VTDR and highlight the need for early detection and tailored interventions to manage DR effectively in younger populations [25].

Clinically, age differences exist in DR. The prevalence of DR in young patients (49%) is much higher than that reported in adults aged 40 years or older (28.5%), with an average diabetes duration of 15 years [21, 26]. In addition, the primary clinical features of VTDR in young patients are active fibrovascular proliferation and progressive TRD [13], which differs from elderly patients, that non-clearing VH accompanied by retinal vascular occlusion are more often detected. Additionally, young patients with PDR have a higher risk of blindness than elderly patients [12]. In align with these findings, young patients in our study have a significantly higher chances of TRD (34.7%) than elderly individuals (16.2%), the same trend was also observed in recurrent VH post-vitrectomy (18.6% vs 10.3%). Interestingly, from our observation, the average AL in young patients of VTDR is 0.9 mm-longer than that of elderly patients. Presumably, AL may play a role underlying the clinical variations of different ages. Through a follow-up of the 736 patients of VTDR, a differential impact on VTDR was observed for patients of different ages. Longer AL is an independent factor preventing TRD for elderly patients, but this beneficial effect was less pronounced in younger patients. Possible attributes for more advanced stage of DR in young patients may include genetic predisposition [27], more undiagnosed diabetes [19], compromised glycemic control due to accelerated decline of β-cell function [19, 28, 29], as well as socioeconomic and psychological burdens. Consequently, scaled-up screenings, early detection and timely intervention are essential steps to tackle DR in young adults. Moreover, social support and psychological help should be reaching these underserved minorities [30, 31].

This comprehensive report initially compared AL and its impact on VTDR across different ages. However, cautions should be taken when considering the generalizability of our findings due to inherent limitations. The key drawback of this study is its retrospective design. Recall bias and high dropout rate might be induced. Second, the prognostic factors of VTDR are multifactorial, however, the factors evaluated in this study were limited for the relatively small sample size of the subgroup in young patients. Nevertheless, the strengths of our study include the longitudinal study design with the rigorous statistical methodology controlling for systemic factors.

In conclusion, young patients with VTDR had significantly longer AL but more aggressive clinical signs with worse prognosis. In elderly group, a longer AL independently reduced the risk of TRD, while this protective effect did not exist for young patients.

## Data Availability

The datasets used and/or analyzed during the current study are available from the corresponding author on reasonable request.

## Abbreviations

DR: Diabetic retinopathy
PDR: Proliferative DR
NPDR: Non- proliferative DR
VTDR: Vision-threatening DR
DME: Diabetic macular edema
TRD: Tractional retinal detachment
NVG: Neovascular glaucoma
VH: Vitreous hemorrhage
ETDRS: Early Treatment Diabetic Retinopathy Study
PRP: Pan-retinal photocoagulation
VEGF: Vascular endothelial growth factor
PPV: Pars plana vitrectomy
BCVA: Best-corrected visual acuity
PVD: Posterior vitreous detachment
DN: Diabetic nephropathy
BMI: Body mass index
HbA1c: Glycated hemoglobin
LDL: Low-density lipoprotein
OR: Odds ratio
CI: Confidence interval
